# Clinical and Metabolic Signatures of *FAM47E*–*SHROOM3* Haplotypes in a General Population Sample

**DOI:** 10.1016/j.ekir.2025.02.018

**Published:** 2025-02-25

**Authors:** Dariush Ghasemi-Semeskandeh, Eva König, Luisa Foco, Nikola Dordevic, Martin Gögele, Johannes Rainer, Markus Ralser, Dianne Acoba, Francisco S. Domingues, Dorien J.M. Peters, Peter P. Pramstaller, Cristian Pattaro

**Affiliations:** 1 Department of Human Genetics, Leiden University Medical Center, Leiden, The Netherlands; 2 Institute for Biomedicine, Eurac Research, Bolzano, Italy; 3 Integrative Data Analysis Unit, Health Data Science Centre, Human Technopole, Milan, Italy; 4 Institute of Biochemistry, Charité - Universitätsmedizin Berlin, Berlin, Germany; 5 Clinical Renal, Late-stage Development, Cardiovascular, Renal and Metabolism, Bio Pharmaceuticals R&D, AstraZeneca, Gothenburg, Sweden; 6 Institut Necker Enfants-Malades, Institut National de la Santé et de la Recherche Médicale U1151, Université Paris Cité, Paris, France

**Keywords:** *CCDC158*, *FAM47E*, haplotype, kidney, *SHROOM3*, *STBD1*

## Abstract

**Introduction:**

Genome-wide association studies (GWAS) identified a locus on chromosome 4q21.1, spanning the Family With Sequence Similarity 47 Member E *(FAM47E)*, Starch Binding Domain 1 (*STBD1)*, Coiled-Coil Domain Containing 158 (*CCDC158)*, and Shroom Family Member 3 (*SHROOM3)* genes, to be associated with kidney function markers. Functional studies implicated *SHROOM3* as the effector gene, demonstrating its developmental role to guarantee podocyte barrier integrity. However, the locus has also been associated with other clinical traits, including electrolytes, hematological, cardiovascular, and neurological traits, not all of which can be easily traced to the regulation of kidney function. We therefore conducted a systematic analysis of the whole locus’ genetic profiles (haplotypes) to assess which phenotypic profiles they were associated with.

**Methods:**

For the 4 genes, we reconstructed haplotypes spanning 71 exonic and intronic variants for 12,834 participants in the Cooperative Health Research in South Tyrol (CHRIS) study based on genotypes imputed on a local whole-exome sequencing (WES) reference panel. Haplotypes were tested for associations with 72 clinical traits, 170 serum metabolites, and 148 plasma protein concentrations, using linear regression models.

**Results:**

We identified 11 haplotypes with a population frequency between 2% and 24%. Compared with the most common haplotype, most haplotypes were associated with higher creatinine-based estimated glomerular filtration rate (eGFR) and lower serum magnesium levels. In addition, specific haplotypes were also associated with biologically diverse groups of traits, including albuminuria, blood pressure, red blood cell traits, carnitines, and amino acids. Cluster analysis highlighted the existence of distinct genetic profiles in which individuals with specific haplotypes presented with specific phenotypic and metabolic signatures.

**Conclusion:**

The genetic variability of the *FAM47E*–*SHROOM3* locus indicates the existence of population subgroups with distinct biomarker profiles.

## Graphical abstract

**Figure:**
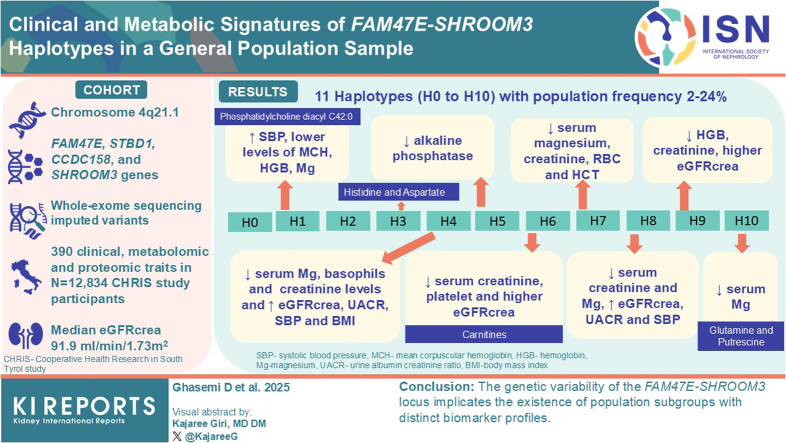

See Commentary on Page 1329

GWAS have consistently identified a genetic locus on chromosome 4q21.1 in prominent association with kidney function markers, including the eGFR, based on serum creatinine (eGFRcrea) or cystatin C^1^, ^2^, ^3^, ^4^, ^5^, ^6^ and albuminuria.^7^ The associated variants were always observed within 2 recombination hotspots (Figure 1a), embedding a high linkage disequilibrium (LD) region (Figure 1b), which encompasses 3 genes (*FAM47E*, *STBD1*, and *CCDC158*) and the first exons of *SHROOM3*.

**Figure 1:**
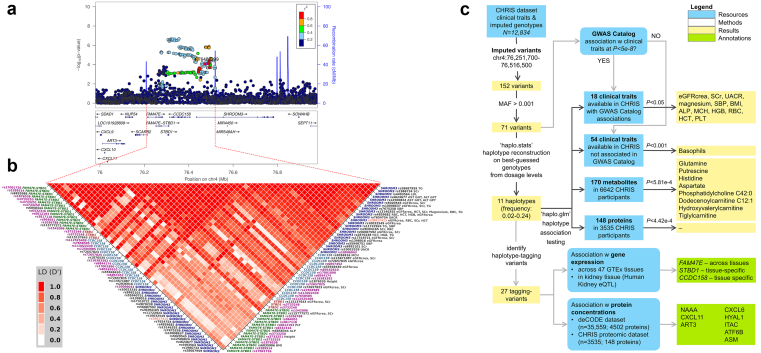
The study locus and the analysis setting. Panel (a). Regional association plot depicting associations between variants in and next to the *FAM47E*–*SHROOM3* locus in the CHRIS study, reflecting a similar association pattern as that identified by a recent CKDGen GWAS meta-analysis^4^: the most associated SNP in the CKDGen analysis is highlighted in purple. SNP positions are referred to the NCBI Build 38. The plot was generated with LocusZoom v1.4.^8^ Panel (b). Linkage disequilibrium pattern of the *FAM47E*–*SHROOM3* locus, based on the D’ statistic. Included are variants associated with complex traits from previous genome-wide association studies and haplotype-tagging variants. The labels on left and right axes are annotated by the corresponding gene symbol and RSID. On the right axis, we also report on the associated complex trait, as per GWAS Catalog interrogation. Color coding of the variants’ IDs indicates: the variant most associated with eGFRcrea^5^ (rs28817415; orange); 25 of the 27 haplotype-tagging variants (2 variants were not present in the LD reference panel; purple); and 31 variants for which there was a GWAS Catalog genome-wide significant association with a trait among those included in the CHRIS study (black). Panel (c). Analysis flowchart. GWAS, genome-wide association study; LD, linkage disequilibrium; RSID, Reference SNP cluster IDentification code; SNP, single nucleotide polymorphism.

Functional studies identified *SHROOM3* as the most likely effector gene. Its encoded protein, Shroom3, regulates cell shape, neural tube formation,^9^ and epithelial morphogenesis. In particular, Shroom3 is involved in mammalian kidney development; it is necessary for developing and maintaining the podocyte architecture,^10^ which is achieved through modulation of the actomyosin network.^11^ During development, lack of Shroom3 causes alterations of the podocyte morphology, glomerular filtration barrier impairment, and glomerular degeneration.^10^ Shroom3 knockdown causes albuminuria and podocyte foot process effacement in mice as well as defective lamellipodia formation in podocytes and disrupted slit diaphragms in rat glomerular epithelial cells.^12^ The risk allele of variant rs17319721, associated with kidney function markers and chronic kidney disease (CKD),^1^^,^^13^ belongs to a genomic sequence that acts as an enhancer of *SHROOM3* expression through transcriptional activation mediated by transcription factor 7 like 2 (*TCF7L2)*.^14^ Altogether, this body of evidence supports *SHROOM3* involvement in CKD.^15^

However, recent evidence shows that *CCDC158*, which seats next to *SHROOM3,* can be implicated in kidney function.^16^ *CCDC158* is involved in renal proximal tubular endocytosis and was found to be expressed in renal proximal tubular cells of young patients with isolated low- and intermediate-molecular-weight proteinuria and nephrocalcinosis, and healthy controls. In controls, but not in patients, *CCDC158* was also expressed in the glomeruli.^16^ Conversely, *STBD1* and *FAM47E* seem to be less directly relevant to kidney function and have broader implications. *STBD1* is a glycogen-binding protein involved in glycophagy, glycogen accumulation, and lipid droplet formation at the lysosomal level.^17^ Owing to its involvement in glucose metabolism, *STBD1* dysregulation may cause cardiometabolic diseases.^17^^,^^18^ *FAM47E* is a protein-coding gene expressed in multiple tissues, which interacts with the protein arginine methyltransferase 5 (*PRMT5*), a multifunctional protein that is critical for the differentiation of primordial germ cells, nerve cells, myocytes, and keratinocytes. *FAM47E*’s role in human physiology and disease is thought to occur through the regulation of *PRMT5* stability, chromatin association, and methyltransferase activity.^19^

It is thus consistent with the functions of involved genes that, beyond kidney function, genetic variants at this locus were associated with diverse phenotypes, including platelets,^20^ hemoglobin (HGB),^20^ serum magnesium,^21^ and Parkinson’s disease.^22^ *SHROOM3* itself was implicated in other, often severe phenotypes, including craniofacial alterations^23^^,^^24^ and cardiac defects.^25^ It is unclear whether these associations share a common root with defects in kidney function. For example, it remains to be clarified whether the apparent mediating role of serum magnesium in the association between eGFRcrea and this locus^26^ reflects real biological mechanisms or joint genetic regulation of the 2 traits.

For these reasons, we investigated the genetic variability of the whole locus, rather than focusing on single variants. The aim of our analysis was to determine which phenotypes occur simultaneously in individuals carrying the same genetic sequence (haplotype) to broaden our understanding of the impact of the whole *FAM47E*–*SHROOM3* locus on human health. We have done so by examining both clinical and molecular aspects, screening 72 clinical traits, 170 target serum metabolites, and 148 plasma protein markers, measured in a large population study conducted on European ancestry individuals, where CKD prevalence is about 9%, like most European countries.^27^ Notoriously, haplotype analysis is not conducted to illuminate causal mechanisms, but it is more helpful to explore genetic heterogeneity in populations.^28^ Moreover, none of the previous WES association studies identified any exonic variant associated with any trait in this locus.^29^, ^30^, ^31^ Nevertheless, to enhance their functional relevance, haplotypes covering the whole *FAM47E*–*SHROOM3* locus were reconstructed based on genetic variants imputed from a WES panel derived from a large subset of the same population study.

We identified common haplotypes that, in addition to sharing association with kidney function markers, were each associated with different phenotypic spectra. With respect to *FAM47E*–*SHROOM3*, cluster analysis supported the existence of subgroups of general population individuals characterized by different clinical and metabolic profiles. Finally, the association with proteins whose encoding genes are located outside the locus further underlines the multifaceted role and involvement of *FAM47E*–*SHROOM3* in human health.

## Methods

### Study Sample

We analyzed data from the Cooperative Health Research in South Tyrol (CHRIS) study, conducted between 2011 and 2018.^32^ Following overnight fasting, 13,393 participants underwent early morning blood drawing, urine collection, anthropometric measurements, and blood pressure measurements.^33^ Health and lifestyle information was gathered through computer-based standardized questionnaire-based interviews. An overview of this analysis is shown in Figure 1c.

### Ethics

The Ethics Committee of the Healthcare System of the Autonomous Province of Bolzano, South Tyrol, approved the CHRIS baseline protocol on April 19 2011 (21-2011). The study conformed to the Declaration of Helsinki and the national and institutional legal and ethical requirements. All participants included in the analysis gave written informed consent.

### Genomics

All DNA samples were genotyped using the Illumina HumanOmniExpressExome or Omni2.5Exome arrays (Illumina, Inc., San Diego, CA) and called with GenomeStudio v2010.3 with default settings on GRCh37, lifted to GRCh38 via CrossMap v0.6.5.^34^ Variants with a GenTrain score < 0.6, cluster separation score < 0.4, or call rate < 80% were considered technical failures and discarded. Variants present on both arrays were subjected to further quality control and removed if monomorphic or not in Hardy-Weinberg equilibrium (*P* < 10^*−*6^). Samples with < 0.98 call rate were removed.

Samples from a subset of 3840 participants underwent WES (xGen Exome Research Panel v1.0; McDonnell Genome Institute, Washington University, MO). After data processing, read alignment, and quality control,^35^^,^^36^ 3422 samples with a post–quality control mean target coverage of 68.4× were used as a population-specific reference panel for genotype imputation onto the whole CHRIS sample as reported previously.^35^ Based on 181 WES samples excluded from the reference panel for testing purposes, we observed excellent agreement between imputed genotypes and sequenced hard-calls (median Pearson’s correlation = 0.99).^35^

### Clinical, Metabolomics, and Proteomics Traits

Information on genotype data and clinical traits was available for 12,834 participants. The clinical traits included blood and urinary markers, diastolic and systolic blood pressure (SBP), and body mass index (Supplementary Table S1). eGFRcrea was estimated with the race-free CKD-Epidemiology Collaboration equation using the R package ‘nephro’ v1.3.0.^37^ Laboratory assay effects^38^ were addressed using quantile normalization as detailed elsewhere.^26^ After removing traits with > 10% missing data, 72 traits remained available for analysis. Missing values were imputed via multiple imputation by chained equations (MICE) using the R package "mice" v3.17.0.^39^ All clinical traits were included in the MICE procedure together with age, biological sex, and the first 10 genetic principal components, to account for population structure. We applied predictive mean matching, performing 3 iterations (maxit = 3) and 150 versions per imputed dataset (m = 150). To create a final complete dataset for use in haplotype reconstruction models, we averaged each trait within each individual. Diagnostic checks of the imputed data demonstrated the plausibility of imputation (Supplementary Figure S1A).

Targeted metabolomic analysis involved a subset of 7252 participants, whose serum samples were analyzed using the AbsoluteIDQ p180 kit (Biocrates Life Sciences AG, Innsbruck, Austria). Normalization and quality control of the 188 measured metabolites are described elsewhere.^40^ To increase sample homogeneity, pregnant women and individuals of non-European descent were excluded. Metabolites with >10% missing data were excluded. This left 170 metabolites available on 6642 samples (Supplementary Table S1). Missing values were imputed using the same approach as that described for clinical traits (diagnostic plots for metabolites with the most missing values are reported in Supplementary Figure 1B).

In a subset of 4087 participants we measured 148 plasma proteins, using mass spectrometry–based scanning SWATH.^41^ Data generation and processing was described elsewhere.^42^ After merging with the genetic data, 3535 participants remained for the analysis (Supplementary Table S1).

### Haplotype Association Analysis

The region of interest on chromosome 4 was bounded by 2 recombination peaks at positions 76,251,700 and 76,516,500, encompassing 152 WES-based imputed variants spanning *FAM47E*, *FAM47E*–*STBD1*, *CCDC158*, and *SHROOM3* (Figure 1a**)**. Retaining 71 variants with minor allele frequency > 0.001 and with imputation quality index Rsq > 0.3 (median Rsq = 0.87; Supplementary Table S2), haplotype reconstruction and regression analysis was conducted using the *haplo.glm* function of the R package’ haplo.stats’ v1.8.9, exploiting an expectation-maximization algorithm for haplotype inference^43^ (Supplementary Methods). Alleles were aligned using the major allele as a reference. Haplotypes with <0.02 frequency were collapsed into a rare-haplotype category. Linear association models were fitted to the inverse normal transformation of each trait, metabolite, and protein, including haplotypes as predictors, and adjusted for age, sex, and the first 10 genetic principal components, estimated on the genotyped autosomal variants to control for population structure.

**Figure 2:**
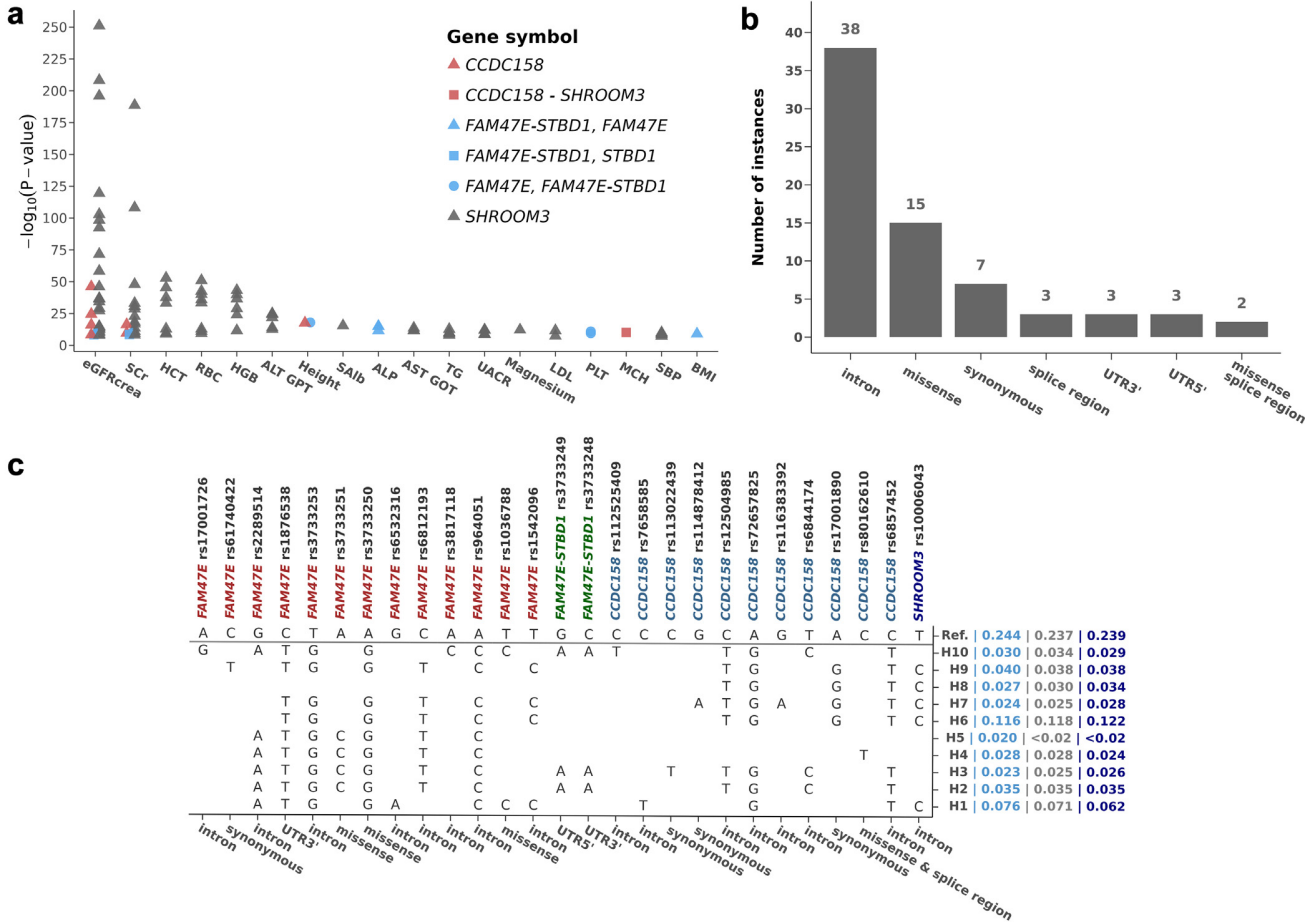
Characteristics of the variants included in the *FAM47E*–*SHROOM3* region on chromosome 4 (76,251,700 - 76,516,500 bp). Panel (a). Associations of the 71 variants used for haplotype reconstruction and 18 GWAS Catalog traits that are also present in the CHRIS study. Reported are the −log_10_(*P*-values) of the association tests, limited to the significant associations. Colors and shapes of the dots are used to distinguish the different genes the variants belong to. The traits are as follows: ALP, alkaline phosphatase; ALT GPT, alanine transaminase; AST GOT, aspartate aminotransferase; BMI, body mass index; eGFRcrea, creatinine-based estimated glomerular filtration rate; HCT, hematocrit; height; HGB, hemoglobin; LDL, low-density lipoprotein cholesterol; MCH, mean corpuscular hemoglobin; PLT, platelet count; RBC, red blood cell count; Salb, serum albumin; SBP, systolic blood pressure; SCr, serum creatinine; serum magnesium; TG, triglycerides; and UACR, urine albumin-to-creatinine ratio. Panel (b). Barplot of the most severe consequences of the 71 variants identified for haplotype reconstruction. Panel (c). Distribution of the 11 reconstructed haplotypes, identified by 27 tagging variants with their functional consequences given on the x-axis. Haplotype frequencies are reported right of the haplotype label for the 3 analyzed subsamples in the order: all individuals with clinical traits; those with also metabolites measurements; those with additional protein measurements. GWAS, genome-wide association study.

Among the 72 considered clinical traits, 18 have been previously reported to be genome-wide significantly associated with variants in the locus (GWAS Catalog interrogation at https://www.ebi.ac.uk/gwas/ on 23-Aug-2023; Figure 2a); these traits were tested for association with the haplotypes at the significance level α = 0.05, considering the strong previous evidence of association. For the remaining clinical traits, the 170 metabolites, and the 148 proteins, we set α at 0.05/46 = 0.001, 0.05/86 = 5.81 × 10^−4^ and 0.05/113 = 4.42 × 10^−4^, respectively, where the denominators were the number of independent principal components necessary to explain 95% of each dataset’s variability. Principal components were estimated with the *prcomp* function in the R package "stats" v4.3.0. This penalization approach was preferred over the more conservative Bonferroni correction, to recognize the presence of substantial correlation structures in the data, particularly between metabolites.

When haplotypes already associated with clinical traits were also associated with a metabolite, we assessed the presence of mediation using Sobel’s test,^44^ at a statistical significance level of 0.05, divided by the number of metabolites.

### Cluster Analysis

We performed hierarchical clustering of the z-scores obtained from significant associations of haplotypes with traits and metabolites. Similarity was based on the Euclidean distance and clustering implemented based on the "Ward D2" approach via *hclust* in the "stats" R package. We applied the Silhouette method to the nonscaled z-scores to determine the optimal number of clusters for haplotypes and traits, using the *fviz_nbclust* and *hcut* functions in the R package "factoextra" v1.0.7, allowing a maximum of 9 and 21 clusters, respectively.

### Variant Annotation

Genetic variants were annotated using Ensembl Variant Effect Predictor v100.2 (http://www.ensembl.org/info/docs/tools/vep/index.html), which predicts the most severe consequences using the "split-vep" plugin. LD between WES-imputed variants selected for haplotype analysis and previously reported common variants associated with the traits of interest was assessed through the D′ statistic, which reflects the underlying haplotype diversity,^45^ estimated using PLINK v1.9.^46^

Out of all 71 variants included in the haplotype reconstruction, we identified the haplotype-tagging variants, that is, the minimal subset of variants that were sufficient to uniquely identify all haplotypes.^47^ We queried the haplotype-tagging variants in the European ancestry datasets of the GTEx Consortium v8 database (https://gtexportal.org/home/; August 10, 2023) across 47 tissues (*n* = 65 to 573 samples per tissue) and in Human Kidney eQTL data^48^ (*n* = 686), to test association with the expression of the 4 genes in the locus (*P* < 5 × 10^−8^), and across 4502 whole blood protein GWAS summary results available^49^ to identify protein quantitative trait loci (pQTLs) at *P* < 5 × 10^−8^ (*n* = 35,559; https://decode.com/summarydata; October 11, 2023).

## Results

### Characterization of the *FAM47E*–*SHROOM3* Genomic Variants and Haplotypes

The 12,834 participants (54.3% females) had a median age of 46 years and median eGFRcrea level of 91.9 ml/min per 1.73 m^2^; 3.6% had eGFRcrea < 60 ml/min per 1.73 m^2^ and 5.9% had urine albumin-to-creatinine ratio (UACR) > 30 mg/g (Table 1). The sample appeared to be an extract of the general population with no particularly prevalent clinical aspects (Supplementary Table S1).

**Table 1:** Main characteristics of the study sample

Participants’ characteristics	Group of traits and sample size		
	Clinical traits (*n* = 12,834)	Metabolites (*n* = 6642)	Proteins (*n* = 3535)
Age, yrs	46 (31–57)	46 (32–58)	46 (32–58)
Females	6969 (54.3%)	3650 (55.0%)	1977 (55.9%)
eGFRcrea ml/min per 1.73 m^2^	91.9 (81.7–104.1)	91.2 (81.1–103.5)	91.2 (80.8–103.3)
eGFRcrea < 60 ml/min per 1.73 m^2^	460 (3.6%)	269 (4.1%)	143 (4.1%)
UACR > 30 mg/g	752 (5.9%)	393 (5.9%)	225 (6.4%)
HbA1c > 6.5%	189 (1.5%)	97 (1.5%)	54 (1.5%)
Hypertension^a^	2023 (15.8%)	1002 (15.1%)	536 (15.2%)

eGFRcrea, creatinine-based estimated glomerular filtration rate; UACR, urine albumin-to-creatinine ratio.

Data are described as median (interquartile range) or number of cases (percentage), as appropriate. Additional characteristics are described in Supplementary Table S1.

a Systolic blood pressure > 140 mmHg or diastolic blood pressure > 90 mmHg.

Within the locus, we identified 71 WES-imputed variants. They were largely intronic, but included missense variants as well as synonymous, splicing, and other types of functional variants (Figure 2b, Supplementary Table S2). The variants were in strong-to-perfect LD with common variants previously associated with common traits (Figure 1b). GWAS catalog interrogation confirmed that the locus was highly pleiotropic (Figure 2a; Supplementary Figure S2).

**Figure 3:**
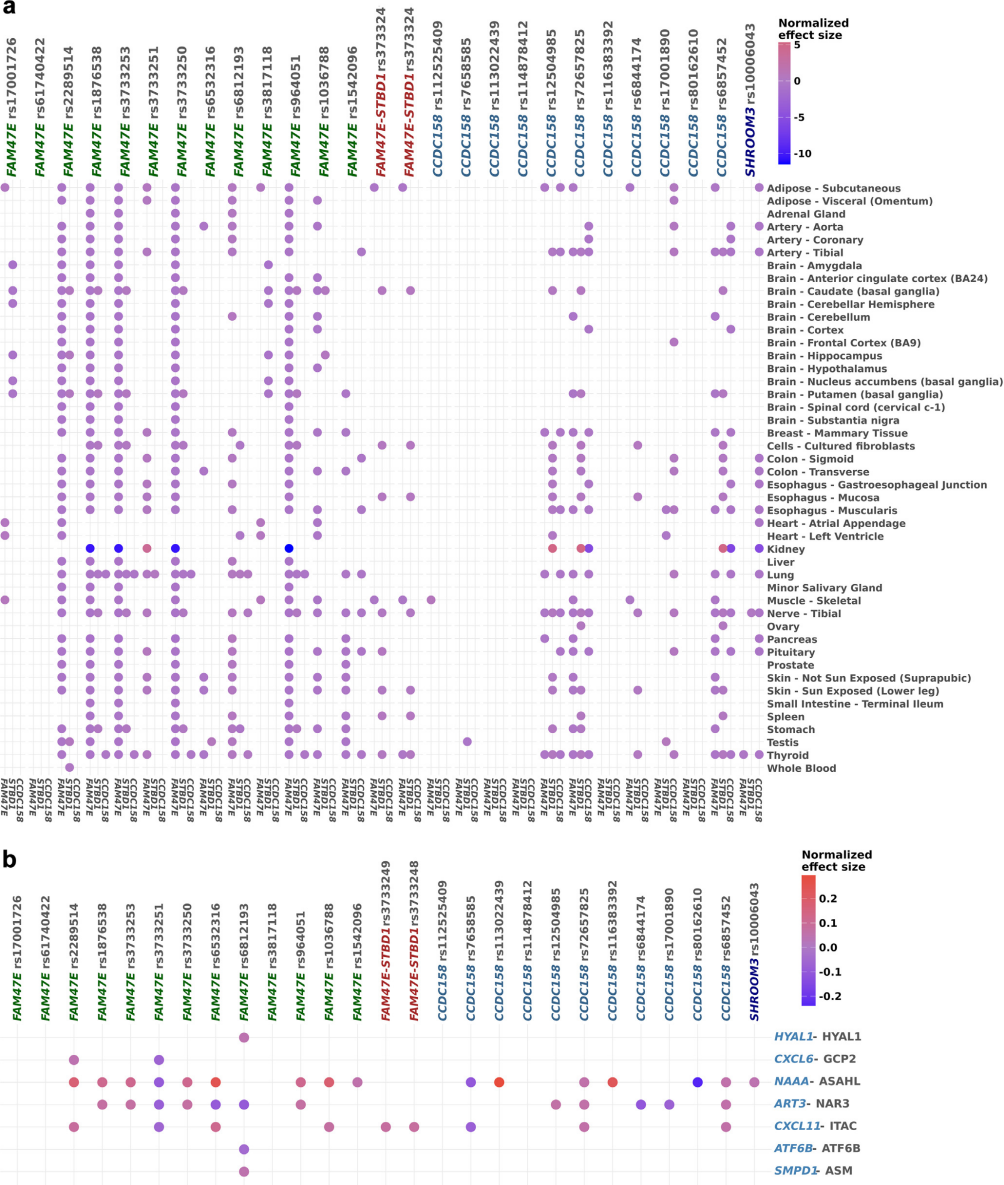
Association of the 27 haplotype-tagging variants with transcriptomic and proteomic levels. Panel (a). Associations between the 27 haplotype-tagging variants and gene expression of *FAM47E*, *STBD1*, and *CCDC158* (horizontal axis; genes grouped by variant) across 45 tissues retrieved from the GTEx v8 dataset (uterus and vagina tissues and *SHROOM3* were omitted for the lack of significant results) and the Human Kidney eQTL data^48^ (vertical axis).The GTEx’s normalized effect sizes are reported. Panel (b) Associations between the 27 haplotype-tagging variants (horizontal axis) and protein concentrations (vertical axis) retrieved from the deCODE dataset.^49^ Dot colors represent the normalized effect size. Only proteins with a significant association with at least one variant are listed.

We identified 11 haplotypes (H0–H10, where H0 was used as the Reference haplotype in all analyses) with ≥ 2% frequency (Supplementary Figure S3), which were uniquely tagged by 27 of the 71 variants (Figure 2c), including 3 *FAM47E* missense variants (rs3733251, rs3733250, and rs1036788) and a splice variant in *CCDC158* (rs80162610). Similar haplotype distributions were observed in the metabolomic and proteomic subsamples. The 27 tagged variants were not associated with *SHROOM3* expression, likely because of the lower expression of *SHROOM3* in adult tissues (Figure 3a; Supplementary Tables S3 and S4). rs2289514; rs1876538; rs3733253; rs964051; and the *FAM47E* missense variant, rs3733250, were associated with *FAM47E* expression in most tissues. The remaining variants were associated with the expression of at least 1 of *FAM47E*, *CCDC158*, and *STBD1* in different tissues. In the Human Kidney eQTL data, *FAM47E* variants rs1876538, rs3733253, rs3733250, and rs964051 were eQTLs for *FAM47E* itself; and variants rs72657825 and rs6857452 in *CCDC158* were eQTLs for *CCDC158* itself (Figure 3a). Given that all 6 variants were eQTLs for the same genes across several tissues, we concluded that none of these variants was kidney-specific for any of the 4 investigated genes (Supplementary Tables S3 and S4).

The proteins encoded by the 4 genes in the locus were not included in the CHRIS or deCODE plasma proteomic datasets.^36^^,^^49^ However, in the latter, we observed associations with proteins (Figure 3b) whose encoding genes were also located on chromosome 4q21.1, immediately to the left of the recombination peak next to *FAM47E*: ADP-ribosyltransferase 3 (NAR3) encoded by *ART3*, N-acylethanolamine acid amidase (NAAA) encoded by the homonymous gene, and C-X-C motif chemokine ligand 11 (CXCL11; the localization is shown in Figure 1a). Twenty-two of the 27 haplotype-tagging variants were associated with at least 1 of the 3 proteins. Specifically, *FAM47E* missense variant rs3733251 was associated with all 3 proteins and the C-X-C motif chemokine ligand 6 (CXCL6), whose encoding gene seats on the contiguous 4q13 cytoband. Other variants associated with all 3 adjacent proteins were rs6532316 in *FAM47E* and rs72657825 and rs6857452 in *CCDC158.* NAAA was associated with most variants, including the *SHROOM3* intronic variant rs10006043. Variant rs6812193 in *FAM47E* was additionally associated with sphingomyelin phosphodiesterase 1 (SMPD1), hyaluronidase 1 (HYAL1), and activating transcription factor 6 beta (ATF6B), whose encoding genes are in different chromosomes.

### Haplotype Association Analyses and Hierarchical Clustering

Haplotypes were first tested for association with the 18 traits for which there were previously reported associations with single variants at the locus (Table 2, Supplementary Table S5). Compared with the reference haplotype, haplotype H1 was associated with higher SBP and lower levels of mean corpuscular hemoglobin (MCH), HGB, and magnesium. H4 was associated with lower serum magnesium and creatinine levels, as well as higher eGFRcrea, UACR, SBP, and body mass index. H5 was associated with lower alkaline phosphatase. H6, the second most common haplotype (frequency = 11.6%), was associated with lower serum creatinine (*P* = 4.92×10^−6^) and platelet count and higher eGFRcrea (*P* = 0.004). H7 was associated with lower serum magnesium and creatinine levels (*P* = 0.044). H8 was associated with lower serum creatinine levels (*P* = 0.001) and higher eGFRcrea (*P* = 2.72 × 10^−4^), UACR (*P* = 0.003), and SBP (*P* = 0.019). H9 was associated with lower HGB, red blood cell count, hematocrit, magnesium, and serum creatinine, and higher eGFRcrea. H10 was associated with lower magnesium levels. Rare haplotypes were associated with height (*P* = 0.021), hematocrit (*P* = 0.008) and HGB (*P* = 0.026). For the 54 remaining traits without previous evidence of association with variants in the locus, we identified an additional association between H4 and lower basophil levels (*P* = 4.18 × 10^−4^), after multiple testing control.

**Table 2:** Statistically significant^a^ haplotype associations with blood, urine, anthropometric, and metabolic traits in the CHRIS study

Group	Haplotype	Trait	Effect (SE)^b^	*P-value*
Clinical traits	H1	SBP	0.056 (0.022)	0.013044
		MCH	–0.063 (0.027)	0.018721
		HGB	–0.044 (0.021)	0.037098
		Magnesium	–0.056 (0.027)	0.040787
	H4	Basophils	–0.145 (0.041)	4.09×10^-4^
		Magnesium	–0.139 (0.041)	6.63×10^-4^
		eGFRcrea	0.089 (0.029)	0.002242
		BMI	0.118 (0.039)	0.002317
		Serum creatinine	–0.094 (0.032)	0.003428
		UACR	0.086 (0.039)	0.027893
		SBP	0.067 (0.034)	0.044951
	H5	ALP	–0.101 (0.049)	0.038823
	H6	Serum creatinine	–0.084 (0.018)	4.34×10^-6^
		eGFRcrea	0.048 (0.017)	0.004067
		PLT	–0.052 (0.023)	0.021421
	H7	Magnesium	–0.106 (0.043)	0.014811
		Serum creatinine	–0.069 (0.034)	0.042162
	H8	eGFRcrea	0.113 (0.031)	2.74×10^-4^
		Serum creatinine	–0.112 (0.034)	0.001064
		UACR	0.124 (0.041)	0.002785
		SBP	0.070 (0.036)	0.047882
	H9	HGB	–0.071 (0.027)	0.008728
		RBC	–0.071 (0.030)	0.016709
		HCT	–0.063 (0.028)	0.025703
		eGFRcrea	0.056 (0.025)	0.026982
		Magnesium	–0.075 (0.035)	0.031865
		Serum creatinine	–0.055 (0.028)	0.047306
	H10	Magnesium	–0.125 (0.040)	0.001786
	Rare	HCT	–0.039 (0.015)	0.008342
		Height	–0.029 (0.013)	0.020452
		HGB	–0.032 (0.014)	0.026074
Metabolites	H1	Phosphatidylcholine diacyl C42:0	0.134 (0.038)	4.39×10^-4^
	H3	Histidine	–0.216 (0.061)	4.06×10^-4^
		Aspartate	–0.214 (0.062)	5.53×10^-4^
	H6	Dodecenoylcarnitine	–0.116 (0.032)	2.71×10^-4^
		Hydroxyvalerylcarnitine^c^	–0.122 (0.032)	1.23×10^-4^
		Tiglylcarnitine	–0.113 (0.032)	4.51×10^-4^
	H10	Glutamine	–0.196 (0.051)	1.08×10^-4^
		Putrescine	–0.194 (0.053)	2.42×10^-4^

ALP, alkaline phosphatase; BMI, body mass index; eGFRcrea, creatinine-based estimated glomerular filtration rate; HCT, hematocrit; HGB; hemoglobin; MCH, mean corpuscular hemoglobin; PLT, platelet count; RBC, red blood cell count; SBP, systolic blood pressure; UACR, urine albumin-to-creatinine ratio.

No significant association was observed with proteins.

a Statistical significance was set at α = 0.05 for traits with previous evidence of association and α = 0.05 to the number of principal components explaining 95% of the set variability for traits and metabolites with no prior evidence of association (see Methods).

b Effects are expressed in terms of standard deviations as all traits were normalized using the inverse-normal transformation (see Methods).

c Also known as Methylmalonylcarnitine.

Haplotypes were associated with serum metabolite (Table 2; Supplementary Table S6); H1 with higher phosphatidylcholine C42:0 (*P* = 4.39 × 10^−4^), H3 with lower histidine (*P* = 4.28 × 10^−4^) and aspartate (*P* = 5.53 × 10^−4^), and H10 with lower glutamine (*P* = 1.08 × 10^−4^) and putrescine (*P* = 2.26 × 10^−4^). H6 was associated with lower dodecenoylcarnitine (*P* = 2.82 × 10^−4^), hydroxyvalerylcarnitine (*P* = 1.31 × 10^−4^), and tiglylcarnitine (*P* = 4.48 × 10^−4^) concentrations. In Supplementary Figure S4, we outline all associations between haplotypes, clinical traits, and metabolites. After multiple testing control, no significant association between haplotypes and the 148 targeted plasma proteins was identified (Supplementary Table S7).

We conducted a mediation analysis on H1, H6, and H10, which, in addition to being associated with clinical traits, were associated with 6 metabolites. We did not observe any evidence of mediation between the metabolites and the clinical traits associated with H1 or H10, indicating independent effects (Table 3). Evidence of partial mediation was observed for H6, where H6 effects on all 3 carnitines were attenuated by serum creatinine or eGFRcrea adjustment, indicating the existence of common pathways.

**Table 3:** Covariate-adjusted haplotype-metabolite association models

Haplotype	Phenotype	Covariate	Effect (SE)	P-value	Sobel’s test P-value
H1	Phosphatidylcholine diacyl C42:0	SBP	0.135 (0.038)	0.000340	0.160976
		HGB	0.134 (0.038)	0.000437	0.192786
		Magnesium	0.133 (0.038)	0.000457	0.551280
		MCH	0.133 (0.038)	0.000458	0.678147
H6	Dodecenoylcarnitine	Serum creatinine	−0.098 (0.031)	0.001719	1.08 ×10^−5^
		eGFRcrea	−0.106 (0.031)	0.000712	0.005440
		PLT	−0.117 (0.032)	0.000226	0.253610
	Hydroxyvalerylcarnitine (Methylmalonylcarnitine)	Serum creatinine	−0.109 (0.031)	0.000544	2.16 ×10^−5^
		eGFRcrea	−0.116 (0.032)	0.000253	0.007243
		PLT	−0.123 (0.032)	0.000107	0.449410
	Tiglylcarnitine	Serum creatinine	−0.097 (0.032)	0.002102	1.46×10^−5^
		eGFRcrea	−0.104 (0.032)	0.001016	0.005921
		PLT	−0.115 (0.032)	0.000327	0.076037
H10	Glutamine	Magnesium	−0.197 (0.051)	0.000104	0.377879
	Putrescine	Magnesium	−0.194 (0.053)	0.000255	0.302043

eGFR, estimated glomerular filtration rate; eGFRcrea; eGFR based on serum creatinine; HGB, hemoglobin; MCH, mean corpuscular hemoglobin; PLT, platelet count; SBP, systolic blood pressure.

Association models between each haplotype and the associated metabolite (Table 2) were adjusted for the traits associated with the same haplotype to assess potential mediation.

### Cluster Analyses of Significant Trait-Haplotype Associations

**Figure 4:**
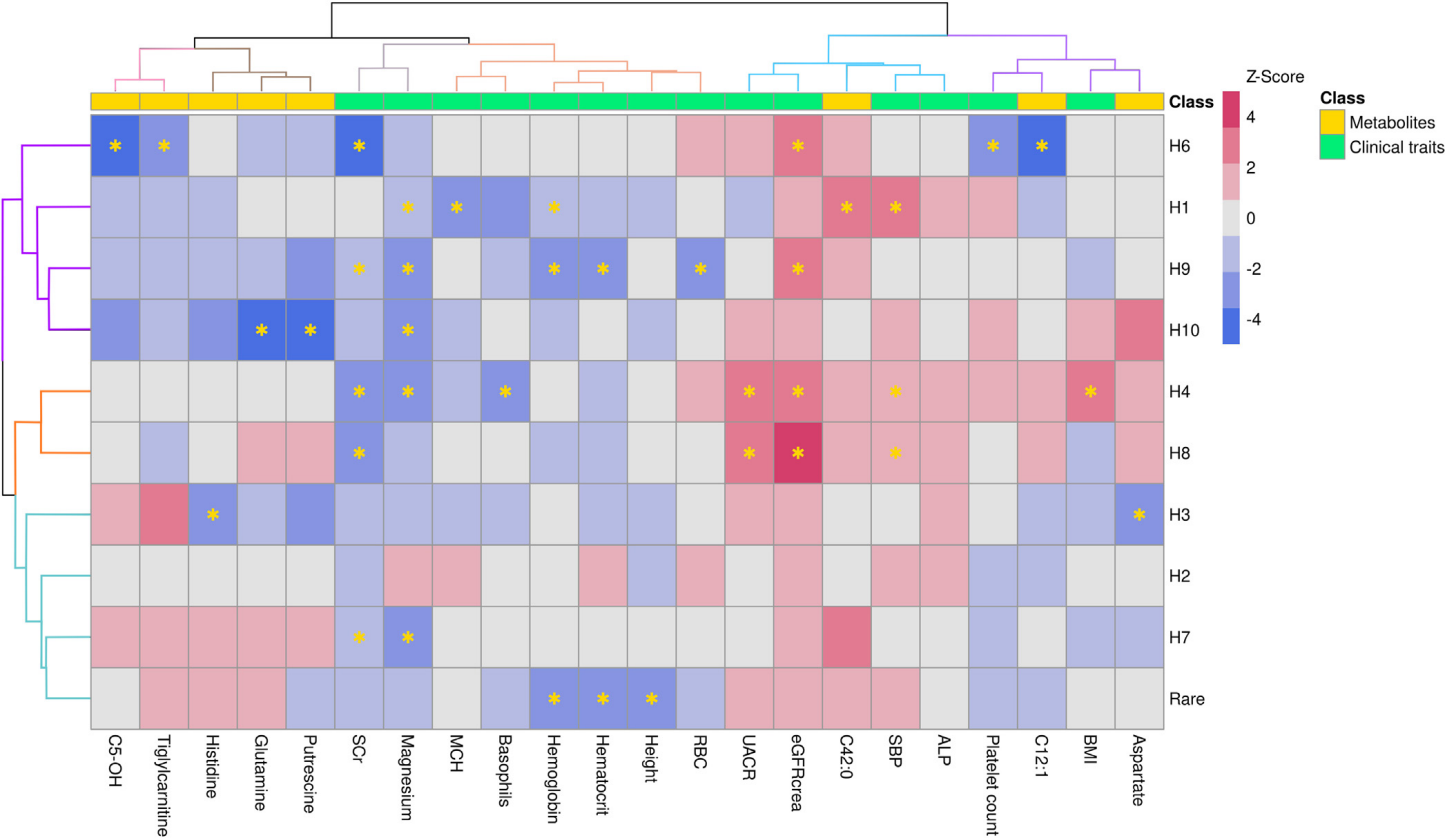
Hierarchical clustering of the associations between haplotypes and any of the 13 clinical traits and 7 metabolites that were associated with at least 1 haplotype (indicated with ∗), in the subset of study participants with metabolites measurements available. H5 was excluded as it did not reach the 2% frequency in this subsample. Clustering was based on the z-scores of association.

Hierarchical clustering of the haplotype effects on the significantly associated clinical traits and metabolites identified 6 clusters of traits, according to the Silhouette method (Figure 4; Supplementary Figure S5). Cluster 1 included a combination of clinical traits (platelet count and body mass index) and metabolites (dodecenoylcarnitine and aspartate levels). Cluster 2 included traits associated with kidney function (eGFRcrea and UACR), blood pressure (SBP), liver transaminases (alkaline phosphatase), and phosphatidylcholine C42:0. Cluster 3 included red blood cells (HGB, hematocrit, red blood cell count, and MCH), basophils, and height. Cluster 4 included the serum creatinine and magnesium levels. Cluster 5 grouped together putrescine, glutamine, and histidine levels. Cluster 6 included carnitines (hydroxyvalerylcarnitine and tiglylcarnitine). When clustering by haplotype, the Silhouette method identified 3 clusters as follows: the first one grouping H1, H6, H9, and H10; the second including H2, H3, H7, and rarer haplotypes; and the third comprising H4 and H8. Altogether, the identified cluster structure suggests the existence of distinct genetic and phenotypic characteristics in *FAM47E*–*SHROOM3*, in which individuals with specific haplotypes present specific phenotypic and metabolic signatures.

## Discussion

Our comprehensive investigation of the *FAM47E*–*SHROOM3* locus highlights the existence of distinct haplotypes that span all genes in the locus and are associated with different clinical and metabolic manifestations.

Most haplotypes were associated with eGFRcrea or serum creatinine, reflecting LD with variants such as rs17319721,^1^ an eQTL for *SHROOM3* through *TCF7L2*-mediated transcriptional activation,^15^ or rs28394165 or rs28817415^5^, which were the most associated with kidney function traits in recent large GWAS.^5^^,^^50^ Specific haplotypes were associated with albuminuria and SBP, some were associated with lower carnitine levels, others were associated with specific amino acids, and others were associated with hematological traits. As shown by cluster analysis, each individual in the population carries specific haplotypes that characterize their clinical and metabolic manifestations. For example, carriers of haplotype H8 would be expected to have higher levels of eGFRcrea, UACR, and SBP than the average population; instead, carriers of H1 or H10 would have lower serum magnesium levels, without necessarily having higher eGFRcrea or UACR; and so on.

Among the common features shared by several haplotypes, we observed a diffuse association with both lower serum magnesium and higher eGFRcrea, suggesting that the previously observed mediation between the two traits^26^ is likely because of a joint regulation by genes in the locus. Association of haplotypes H4 and H8 with lower serum creatinine and higher UACR and eGFRcrea at the same time, is consistent with reduced muscle mass causing lower circulating creatinine and thus higher eGFRcrea and lower urinary creatinine excretion increasing UACR. Nevertheless, H8 association with higher SBP is consistent with the increased risk of incident hypertension in albuminuric individuals.^51^ In fact, both eGFRcrea and UACR can be high in the presence of hyperfiltration following comorbidities such as hypertension,^52^ hyperuricemia,^53^ and diabetes.^54^ Similar results were observed for H4, despite nonsignificant association with higher SBP. Given that H4 and H8 do not share any alleles at the 27 tagging variants, their similar characteristics might reflect LD with functional variants outside the reconstructed haplotypes.

Haplotype H6 was associated with lower serum creatinine, acylcarnitine, dodecenoylcarnitine, hydroxyvalerylcarnitine, and tiglylcarnitine concentrations. Acylcarnitines transport fatty acids from the cytosol into the mitochondria to produce energy through beta-oxidation.^55^ They are freely filtered by the kidney and excreted in the urine.^56^ As kidney function decreases, serum acylcarnitines should increase.^57^ This was observed in patients with CKD exhibiting high serum dodecenoylcarnitine, hydroxyvalerylcarnitine and tiglylcarnitine concentrations linked to low eGFR.^56^ This aligns with our findings, suggesting that H6 might confer nephroprotection through carnitine reduction. This mechanism would be consistent with the observed mediatory effect of eGFRcrea and serum creatinine levels on the association between all carnitines and H6. Alternatively, H6 might be in LD with *SHROOM3* alleles conferring sustained structural integrity to podocytes, resulting in better filtration capacity and lowering both creatinine levels and free acylcarnitine concentrations.

The observed effects of H3 and H10 on amino acid levels (histidine, aspartate, glutamine, and putrescine) might indirectly reflect the role of *STBD1*, which transports glycogen to the lysosomes for breakdown into glucose. Glucose then enters glycolysis, impacting the levels of amino acid-derived metabolites entering or leaving the citrate cycle, which is the core of human metabolism regulation. For example, the association of H3 with both histidine and aspartate concentrations reflects the connection between the 2 amino acids, where histidine is transformed into glutamate, which eventually enters the citrate cycle, leading among others to aspartate. Glutamine, the most abundant amino acid in humans, is a fundamental precursor of glutathione. Under fasting or starvation, glutamine serves gluconeogenesis, helping the liver to maintain blood glucose levels following glycogen-store shortages.^58^ In rats, glutamine supplementation protects against STZ-induced renal injury and prevents downregulation of the kidney injury molecule-1, neutrophil gelatinase-associated lipocalin, TGF-β1, and collagen-1 mRNA expressions.^59^ In patients with diabetes, glutamine supplementation decreases glycemia through increased glucagon-like peptide 1 secretion.^60^ However, the absence of associations of H3 and H10 with clinical traits limits further interpretation. Similarly, haplotype H9, which is associated with higher eGFRcrea and lower levels of red blood cell traits; and H1, also associated with lower red blood cell trait levels, present results that are difficult to interpret without further functional experiments.

We modeled haplotypes included within 2 recombination hotspots at positions 76,251,700 and 76,516,500 on chromosome 4q21.1, which clearly delineated the genetic locus associated with eGFR in all GWAS reported to date. Prokop *et al.*^15^ showed that variants within those 2 recombination peaks are in LD with variants outside peaks such as *SHROOM3* P1244L, located downstream of the second peak and associated with high CKD risk in Eastern Asia.^15^ They also observed that the locus is associated with *TCF7L2*, a chromosome 10 transcriptional factor with a broad phenotypic spectrum.^61^ Our annotation analyses extend these observations, showing that several haplotype-tagging variants are associated with proteins (NAR3, CXCL11, and NAAA) whose encoding genes are located immediately upstream of the recombination hotspot adjacent to *FAM47E*, between chromosome 4 positions 75,910,655 and 76,114,048. These associations may reflect underlying longer haplotypes spanning these genes. NAR3 is encoded by *ART3*, which is specifically expressed in the mesangium of the glomerulus.^62^ This is relevant given that a recent GWAS reported an association between the eGFRcrea and the rs6532204 variant next to *ART3*.^48^ It warrants further investigation to determine whether a joint involvement of *SHROOM3* on podocytes and *ART3* on the mesangium is possible and if that might implicate relevant kidney phenotypes. *CXCL11* is a proinflammatory chemokine implicated in kidney disease induced by interferon signaling.^63^ Urine CXCL11 correlates with diabetic kidney disease progression^64^ and is upregulated in the glomeruli of nephrotic syndrome patients carrying *APOL1* high risk variants.^65^ In mice with acute glomerular inflammation, genetic deletion of *CXCL11* receptor *Cxcr3* attenuates glomerulosclerosis and albuminuria.^66^ NAAA is a proinflammatory protein emerging as a promising target in mouse models of parkinsonism.^67^ Other associated proteins, encoded by genes in other chromosomes (*SMPD1*, *HYAL1*, *ATF6B*, and *CXCL6*), may reflect either *FAM47E* transcriptional activity or biological consequences of the proteins encoded by the genes tagged by the haplotypes.

The main strength of our analysis was the availability of WES data from a subsample that we used to impute exonic variants in the entire study sample of > 12,000 individuals, enabling the reconstruction of haplotypes with sample frequencies as low as 2% and their association testing against clinical traits, metabolites, and proteins. This analysis had some limitations. First, the proteomics panel included few, highly abundant plasma proteins, none of which were associated with haplotypes at this locus. The successful single-variant query of external proteomic datasets suggests that haplotype associations with these proteins might be identified as individual-level data become available. Second, despite the large sample size, haplotypes are multicategory variables that easily generate data sparseness, eroding statistical power. Third, consistent with GWAS studies that identified significant associations with complex traits at this locus, we focused our investigation on the region bounded by the 2 recombination hotspots at chromosome 4 positions 76,251,700 and 76,516,500; given that most *SHROOM3* exons fall outside this segment, this constraint has probably limited the possibility to contextualize *SHROOM3* with the other genes. Conversely, extrapolating haplotypes at arbitrary distances outside the borders would have increased data sparseness. Fifth, considering that we analyzed WES-based data, when most population-based studies are focused on genotyping arrays, the generalizability of the identified haplotypes to a broader European ancestry context should be assessed based on similar genomic platforms or sequencing. Lastly, despite their complexity and having identified the presence of heterogeneous molecular and clinical profiles in the population, our analyses should be considered preliminary to additional fundamental genetic analyses of DNA motifs, transcription binding sites, CHIP-seq, or other types of high-throughput chromosome conformation capture data, which could more exhaustively inform the transcriptional relevance of identified haplotypes. Given that our broad-spectrum investigation has identified clinical and molecular markers of different natures, one difficulty in such analyses would be to identify the relevant tissues and cell types among the many possible ones.

In conclusion, our investigation revealed distinct genetic profiles of *FAM47E*–*SHROOM3* associated with heterogeneous phenotypic and metabolic combinations that warrant further investigation.

## Disclosure

CP received consultancy fees from Quotient Therapeutics. DA was employed at AstraZeneca. All the other authors declared no competing interests.

## Supplementary Material

Supplementary File (PDF) **Supplementary Methods**. **Figure S1.** Diagnostics plot evaluating accuracy of MICE imputation. **Figure S2.** Interrogation of the 27 tagging variants in association with any complex trait in the GWAS Catalog. **Figure S3.** Haplotypes reconstructed based on the 71 available WES-imputed variants. **Figure S4.** Haplotype association analysis results. **Figure S5.** Cluster analysis of the standardized effects of haplotypes on significant clinical traits and metabolites.

Supplementary File (xlsx) **Table S1.** Distribution of the study traits, metabolites, and proteins. **Table S2.** Characteristics of the variants used for haplotype reconstruction in the CHRIS. **Table S3.** Expression QTL variants from GTEx. **Table S4.** Query of 27 haplotype-tagged variants in the kidney eQTL meta-analysis. **Table S5.** Association between haplotypes and clinical traits. **Table S6.** Association between haplotypes and serum metabolites in a subset of 6641 participants. **Table S7.** Association between haplotypes and plasma proteins in a subset of 3535 study participants.

## Footnotes

Supplementary File (PDF) and (xlsx)
**Supplementary Methods**. **Figure S1.** Diagnostics plot evaluating accuracy of MICE imputation. **Figure S2.** Interrogation of the 27 tagging variants in association with any complex trait in the GWAS Catalog. **Figure S3.** Haplotypes reconstructed based on the 71 available WES-imputed variants. **Figure S4.** Haplotype association analysis results. **Figure S5.** Cluster analysis of the standardized effects of haplotypes on significant clinical traits and metabolites. **Table S1.** Distribution of the study traits, metabolites, and proteins. **Table S2.** Characteristics of the variants used for haplotype reconstruction in the CHRIS. **Table S3.** Expression QTL variants from GTEx. **Table S4.** Query of 27 haplotype-tagged variants in the kidney eQTL meta-analysis. **Table S5.** Association between haplotypes and clinical traits. **Table S6.** Association between haplotypes and serum metabolites in a subset of 6641 participants. **Table S7.** Association between haplotypes and plasma proteins in a subset of 3535 study participants.
