# Supplementary material for: Clinical and Metabolic Signatures of FAM47E–SHROOM3 Haplotypes in a General Population Sample
Source: Kidney Int Rep. 2025 Feb 25;10(5):1495–508. doi: 10.1016/j.ekir.2025.02.018 (PMC12142803; doi:10.1016/j.ekir.2025.02.018)

Supplementary materials

Supplementary Methods

Haplotypes were reconstructed using the expectation-maximization algorithm implemented in the R package ‘haplo.stats’ v1.8.9 with the following parameter setting: n.try=2 (number of times to try to maximize the log-likelihood); insert.batch.size=2 (number of loci to be inserted in a single batch); max.haps.limit=4e6 (maximum number of haplotypes for the input genotypes); and min.posterior=1e-5 (minimum posterior probability for a haplotype pair, given the input genotypes).

Supplementary Figure S1. Diagnostics plot evaluating accuracy of MICE imputation. **Panel A**. Density plot of actual (real) clinical traits data against density plot of imputed data averaged over 150 versions of imputed datasets (**see methods**). Among the imputed clinical traits with missing over 2%, the first 10 ones are portrayed here. **Panel B**. Density plot of actual metabolites data against density plot of imputed data averaged over 150 versions of imputed datasets (**see methods**). Among imputed metabolites with missing over 1%, the first 10 ones are depicted here.

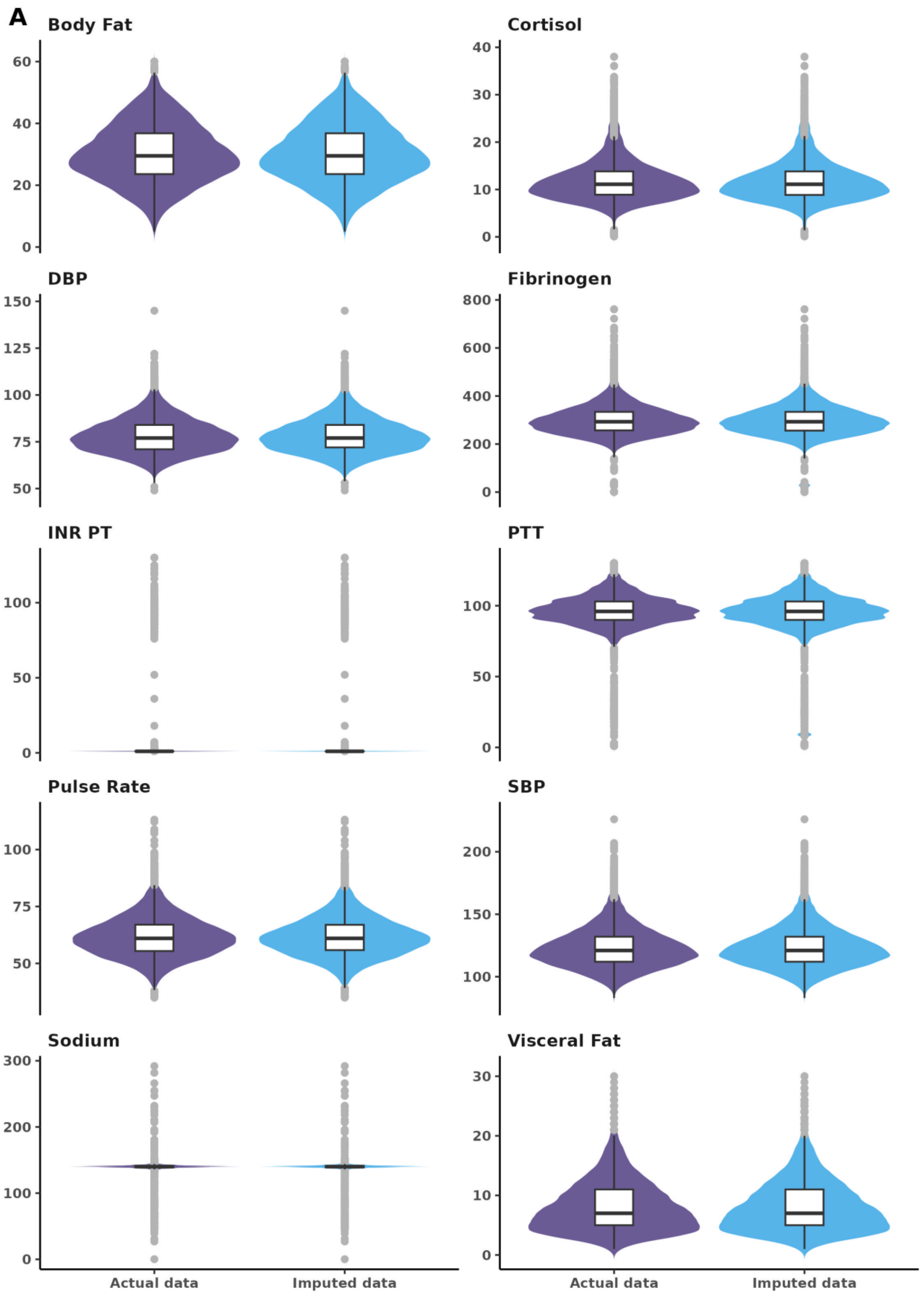
A
Body Fat
Cortisol
DBP
Fibrinogen
INR PT
PTT
Pulse Rate
SBP
Sodium
Visceral Fat
Actual data
Imputed data
Actual data
Imputed data

B

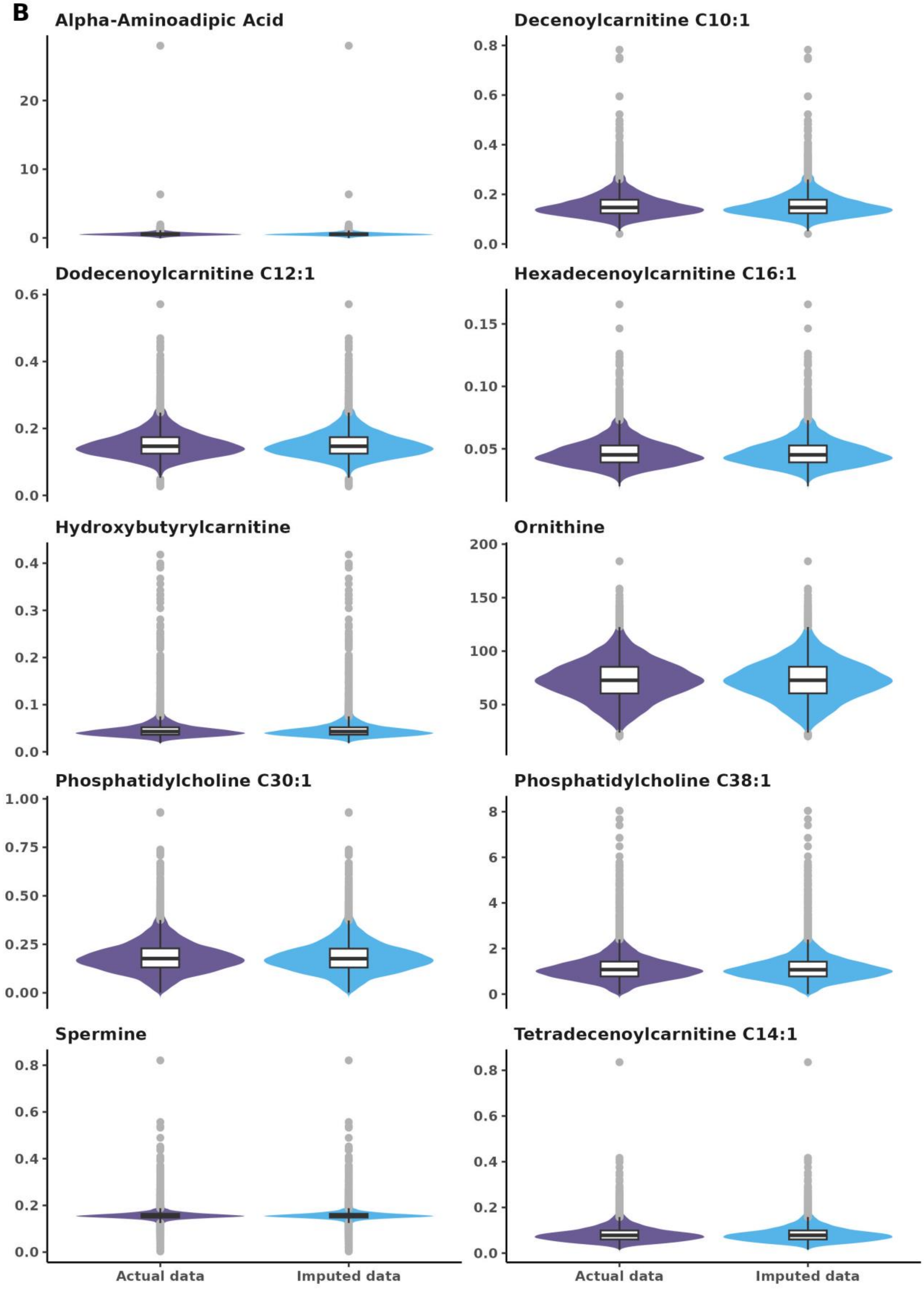

Alpha-Aminoadipic Acid
Decenoylcarnitine C10:1
Dodecenoylcarnitine C12:1
Hexadecenoylcarnitine C16:1
Hydroxybutyrylcarnitine
Ornithine
Phosphatidylcholine C30:1
Phosphatidylcholine C38:1
Spermine
Tetradecenoylcarnitine C14:1
Actual data
Imputed data
Actual data
Imputed data

Supplementary Figure S2. Interrogation of the 27 tagging variants in association with any complex trait in the GWAS Catalog. Y-axis: $-\log_{10}$(P-values) of genome-wide significant associations (P-value<5×10^{-8}). Colors and shapes of the dots are used to distinguish the different genes to which the variants belong.

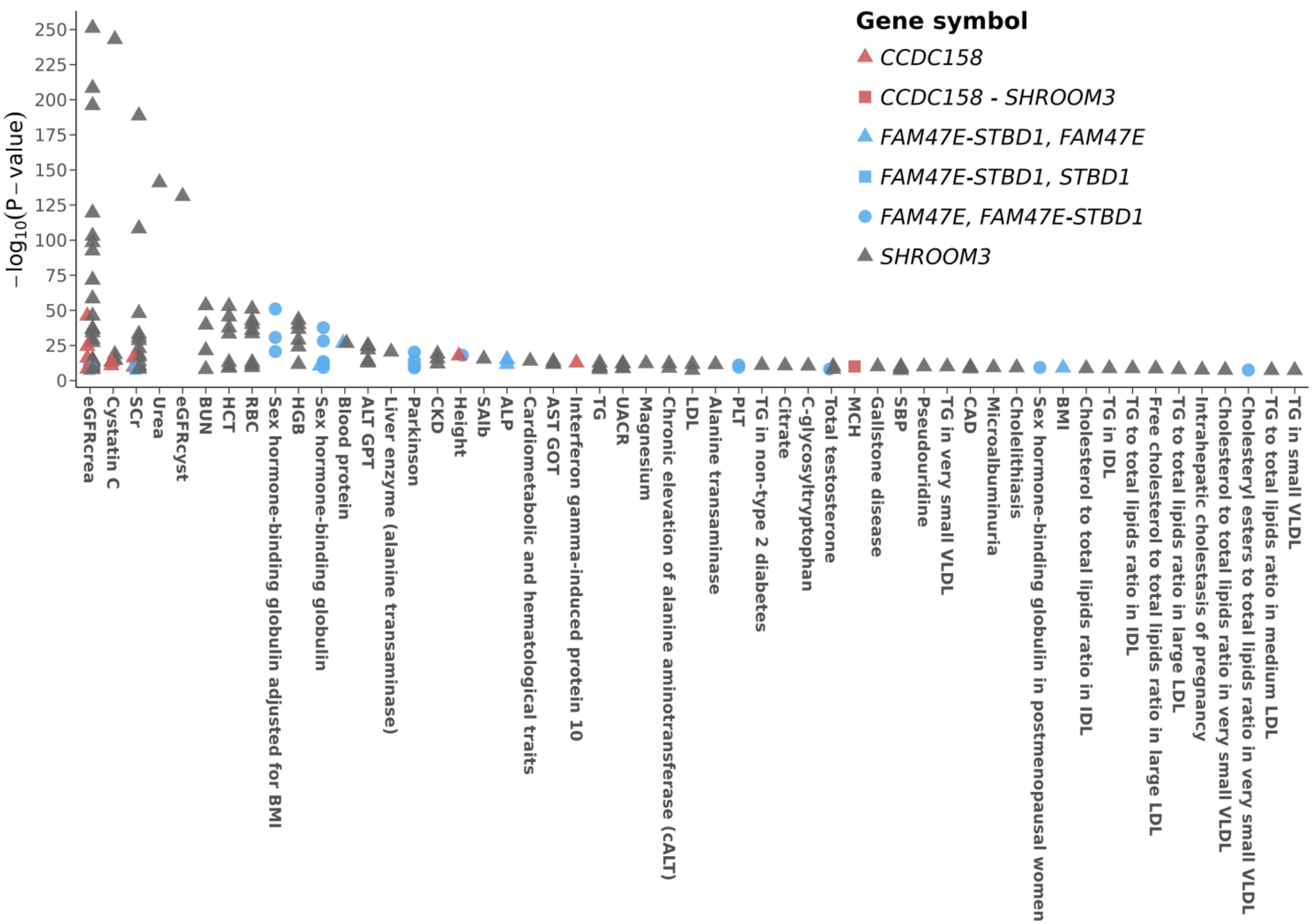

Supplementary Figure S3. Haplotypes reconstructed based on the 71 available WES-imputed variants. On top, variants are listed by gene name – chromosomal position – alleles. At the bottom, variants' functional consequences. Middle panel: the most common, reference haplotype is provided in its entirety (first line). Other haplotypes are presented by listing only the alleles that are different from the reference haplotype. When the allele is the same as in the reference haplotype, the letter is not displayed.

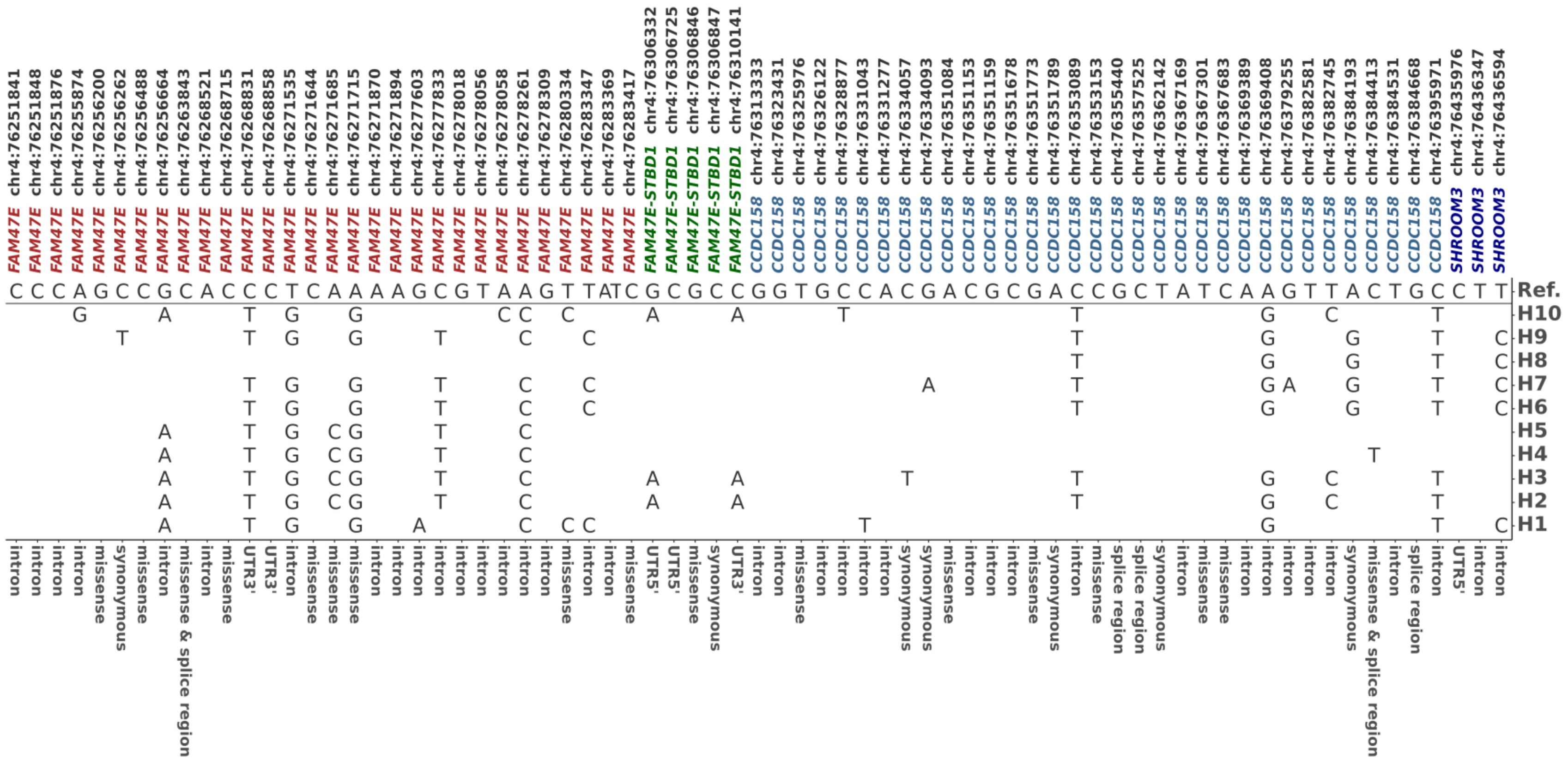

Supplemental Figure S4. Haplotype association analysis results. Displayed are the effect coefficients and their 95% confidence intervals from the associations between haplotypes with the 13 clinical traits and 7 metabolites that were associated with at least one haplotype, in the subset of participants with metabolites measurements available. Haplotype 5 (H5) was excluded as it did not reach the 2% frequency in this subsample.

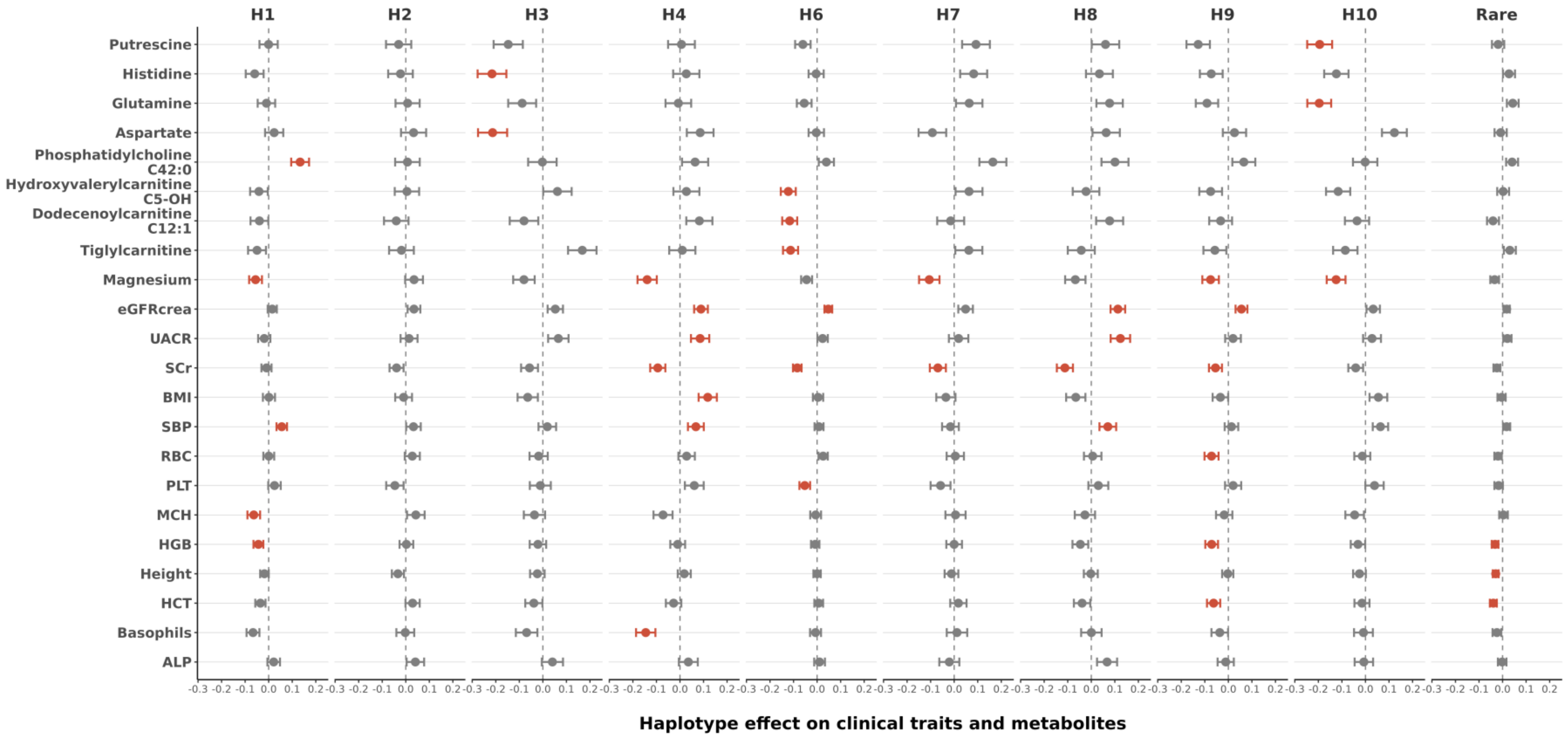

Supplemental Figure S5. Cluster analysis of the standardized effects of haplotypes on significant clinical traits and metabolites. **Panel A**. Results of the Silhouette method applied to the clustering of haplotypes: the number of clusters (x-axis) is plotted against the average silhouette width (y-axis). **Panel B**. Results of the Silhouette method applied to the clustering of traits: the number of clusters (x-axis) is plotted against the average silhouette width (y-axis). **Panel C**. Hierarchical clustering of haplotypes (listed and clustered on the y-axis). **Panel D**. Hierarchical clustering of clinical traits and metabolites (listed and clustered on the y-axis). Colors are used to identify the clusters.

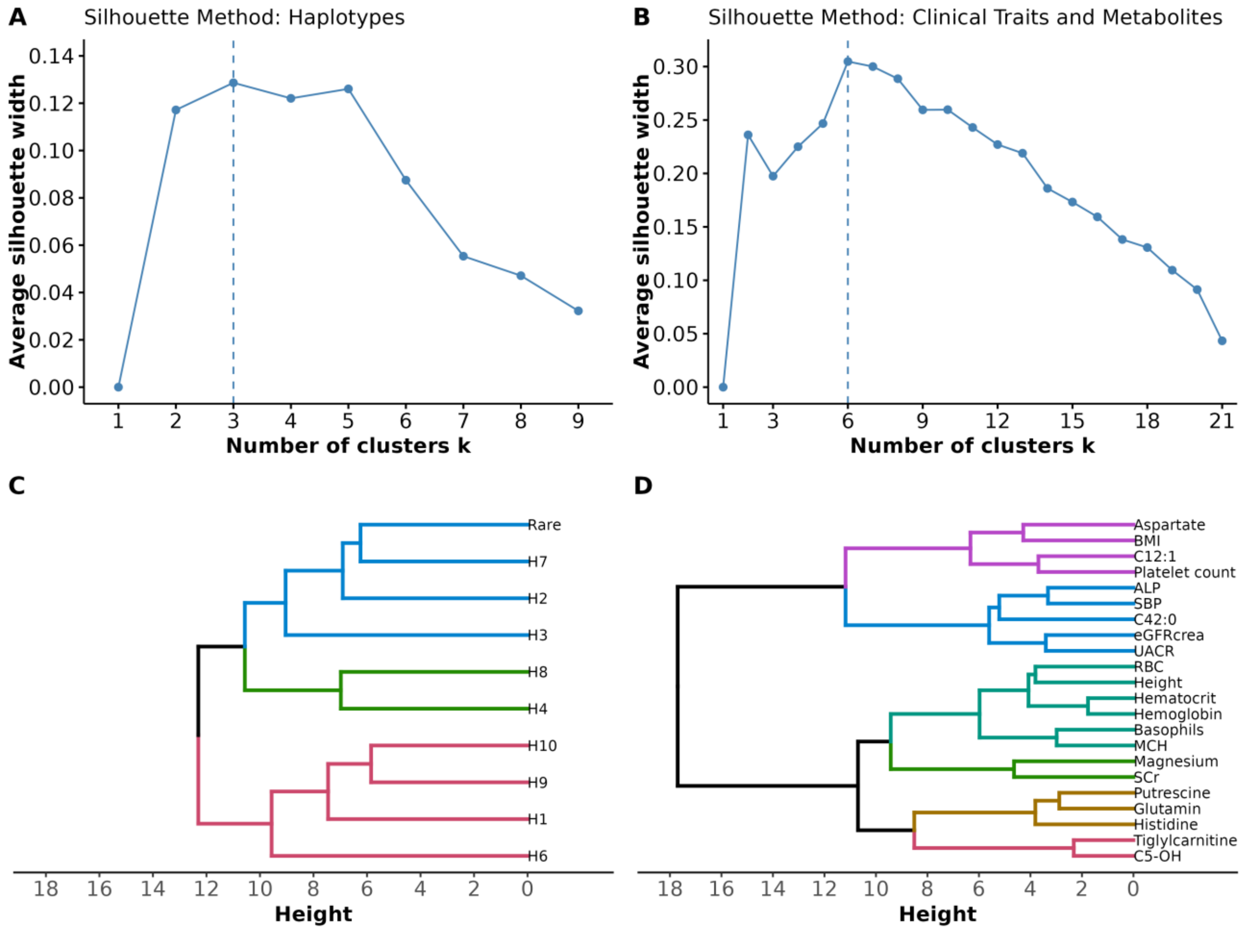

Supplement: Supplementary File (PDF) — Supplementary Methods. Figure S1. Diagnostics plot evaluating accuracy of MICE imputation. Figure S2. Interrogation of the 27 tagging variants in association with any complex trait in the GWAS Catalog. Figure S3. Haplotypes reconstructed based on the 71 available WES-imputed variants. Figure S4. Haplotype association analysis results. Figure S5. Cluster analysis of the standardized effects of haplotypes on significant clinical traits and metabolites. [file mmc1.pdf]
